# Dermoscopy as a Noninvasive Diagnostic Modality in Erythromelanosis Follicularis Faciei et Colli: A Case Series

**DOI:** 10.5826/dpc.1102a20

**Published:** 2021-03-08

**Authors:** Shagufta Rather, Aaqib Aslam Shah, Faizan Younus Shah

**Affiliations:** 1 Department of Dermatology, Government Medical College, Srinagar, University of Kashmir, Jammu and Kashmir, India

**Keywords:** erythromelanosis follicularis faciei et colli, noninvasive, dermoscopy

## Introduction

A myriad of conditions are diagnosed clinically in dermatology without laboratory confirmation, owing to the benign nature of these conditions and lack of a definitive noninvasive diagnostic tests. These conditions can pose a diagnostic dilemma and difference of opinion among clinicians. Erythromelanosis follicularis faciei et colli (EFFC) is one of these rare conditions that may be difficult to pick up especially at early stages. The condition was described for the first time by Kitamura et al and is clinically characterized by the presence of well-demarcated reddish brown patches of hyperpigmentation and erythema along with follicular papules present on the face, extending down to involve the neck [1,2]. It is commonplace to confuse the condition with analogous pigmentary erythematous disorders, resulting in inappropriate treatment protocols with minimal results. Moreover, patients are often reluctant to have an invasive procedure such as skin biopsy owing to the benign nature of this disease and cosmetic concerns, as it involves the face. Dermoscopic diagnosis of the disease can help overcome such a diagnostic quandary. We report clinico-epidemiological profiles and dermoscopic findings in a series of 8 patients presenting with clinical features suggestive of EFFC.

## Case Presentations

Eight consecutive patients with features suggestive of EFFC presented to the outpatient department and were enrolled and subjected to relevant history and clinical examination after taking informed consent. Criteria for diagnosing EFFC included the presence of hyperpigmentation, erythema, and follicular papules involving mainly the face extending to sides of the neck. Patients who met the clinical criteria were subjected to dermoscopic examination using a handheld dermoscope (DermLite DL4; 3Gen, USA; ×10). Histopathological confirmation was performed in cases where diagnosis was doubtful.

**Figure 1:**
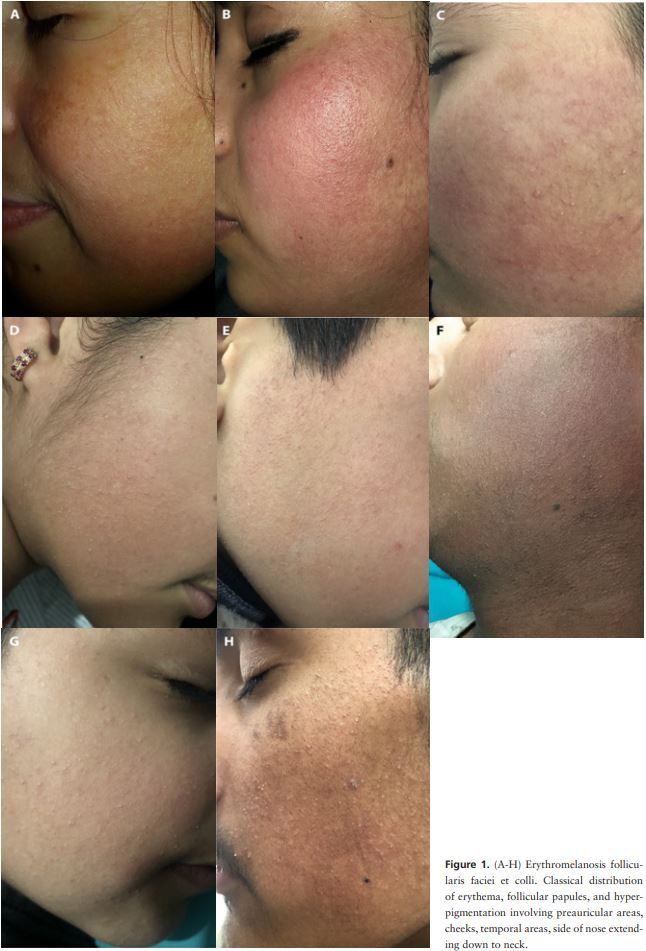
(A–H) Erythromelanosis follicularis faciei et colli. Classical distribution of erythema, follicular papules, and hyperpigmentation involving preauricular areas, cheeks, temporal areas, side of nose extending down to neck.

The clinico-demographic profile and dermoscopic findings of the patients are summarized in Table 1. The ages of the patients ranged from 11 to 24 years with a mean age of 16.1 years. No male or female predominance was seen. Disease onset was between 7 and 13 years of age and mean duration varied from 2 to 10 years. There was history of mild to moderate photosensitivity and xerosis or granular feel. Clinical examination revealed the classical triad of erythema, pigmentation, and follicular papules in all patients while telangiectasias were seen in only 4 cases. Cheeks were the most common site of involvement, followed by preauricular areas and extension of lesions to temporal areas in 6 patients and the submandibular area and neck in 5 patients. Follicular papules over the forehead and ear helices were observed in 2 patients (Figure 1, A–H). Keratosis pilaris, presenting as multiple acuminate keratotic papules with perifollicular erythema was present on the back, upper arms, and outer aspect of thighs in all patients, and 3 of them had positive family history for the same.

**Figure 2:**
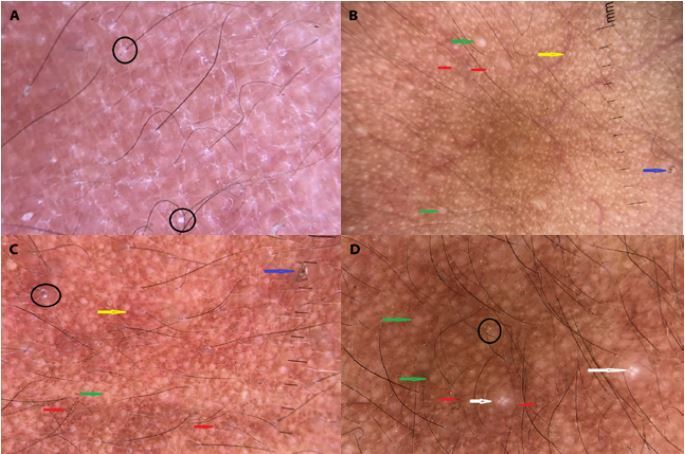
(A–D) Dermoscopy of erythromelanosis follicularis faciei et colli. Numerous follicular keratotic plugs, over a reddish brown background (green arrows). Whitish scales, mainly perifollicular in distribution (black circles). Perifollicular and interfollicular gray-brown dots and granules (peppering) (red arrows). Telengiectasias (yellow arrows). Rosettes (white arrows). Twisted/coiled hair (blue arrows). (DermLite DL4, polarized; original magnification ×10)

**Figure 3:**
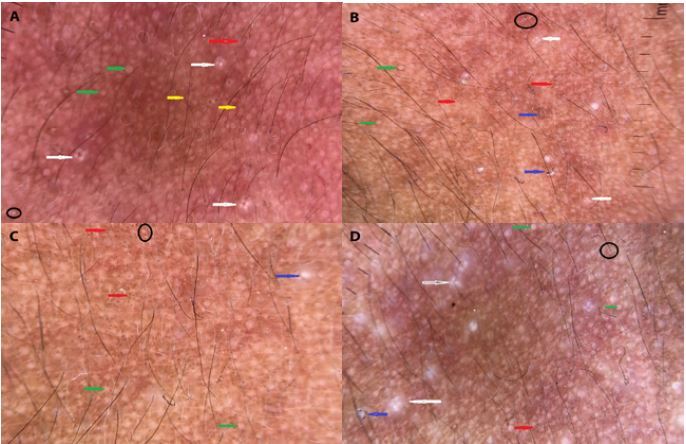
(A–D) Dermoscopy of erythromelanosis follicularis faciei et colli. Numerous follicular keratotic plugs over a reddish brown background (green arrows). Whitish scales, mainly perifollicular in distribution (black circles). Perifollicular and interfollicular gray-brown dots and granules (peppering) (red arrows). Telengiectasias (yellow arrows). Rosettes (white arrows). Twisted/coiled hair (blue arrows). (DermLite DL4, polarized; original magnification ×10).

Dermoscopic examination revealed whitish scales and numerous follicular keratotic plugs against a reddish brown background in all the patients. Gray-blue dots and granules present in the perifollicular and interfollicular areas were seen in most of our patients, especially in subjects with longer duration of disease. Telengiectasias were present in 5 patients. White shiny structures (rosettes) were observed under polarized light in 4 patients. A coiled-up or twisted hair retained inside a follicular prominence with an inflamed follicular papule overlying it was observed in all patients (Figure 2, A–D and Figure 3, A–D).

**Figure 4:**
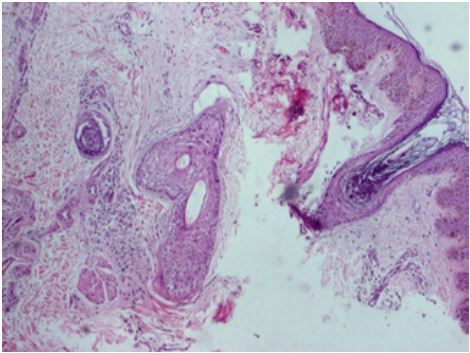
Hyperkeratosis, dilated infundibula with follicular plugging, increased basal layer pigmentation with mild perivascular and periadenaxal chronic inflammatory infiltrate (H&E, ×4).

Histopathology was performed in only 3 cases and revealed hyperkeratosis, dilated infundibula with follicular plugging, and increased pigmentation in the basal membrane along with a mild perivascular and periadnexal chronic inflammatory infiltrate (Figure 4).

## Discussion

EFFC is a rare disorder with limited cases reported across the globe. Multiple contributing etiologies have been proposed [3]. Many studies have also suggested a heredity component in pathogenesis [2,3]. The condition has preponderance in males but occurrence in females has also been reported [4]. No gender predominance was observed in our study. The onset of disease in all our patients was in early childhood, bearing resemblance to other studies in the past [4,5].

The dermoscopic finding of EFFC was reported by Errichetti et al for the first time and our findings were consistent with their study [6]. Mouni et al [7] later on revealed similar findings in their patients. Similar dermoscopic findings can be seen in erythrosis pigmentosa peribuccalis which is considered to be a variant of this disease by few authors [8]. EFFC can be differentiated from many close differentials on dermoscopy. Dermoscopy of melasma reveals light to dark brown uniform patches with capillary network while brownish pseudonetwork along with gray dots and granules with telangiectatic vessels seen in Reihl melanosis [8,9]. Keratosis pilaris rubra atrophicans faciei displays whitish follicular plugs with reddish background with telangiectatic vessels being observed at times, and in poikiloderma of Civatte, structureless brownish pigmentation and telangiectatic vessels can be demonstrated [6]. The findings on dermoscopy of follicular plugging, scaling, and reddish brown background correspond to histopathological findings of hyperkeratotic hair follicles, orthokeratosis, and ectatic vessels respectively. Gray-blue dots and granules correlate with the presence of pigmentary incontinence and dermal melanophages in the upper dermis respectively [6,7]. White rosettes, though encountered in a few cases, are not a specific finding and have been reported in many dermatological disorders like basal cell carcinoma, melanomas, actinic keratosis, seborrheic keratosis, and lichen planus like keratosis [10]. In conditions where there is hair follicle and perifollicular involvement, it has been suggested that the optical effect between polarized light and follicular structures at the infundibular keratin layer leads to formation of rosettes [10]. Narrowing of infundibula or blockage by keratin has been postulated to result in rosette formation by some authors, while others proposed that rosettes correspond to an alternating focal hyperkeratosis and normal corneal layer and keratin-filled acrosyringeal openings [11]. Since EFFC is predominantly a follicular disorder, occurrence of rosettes remains well explained.

So far, no treatment of EFFC has proven satisfactory. Various options that have been explored include topical keratolytic agents such as ammonium lactate (12%), tretinoin cream (0.05%, 1%), salicylic acid 2%, metronidazole, topical tacalcitol and hydroquinone 4%. Intermittent oral isotretinoin, chemical peel, and long-pulsed dye laser are other treatment options being considered [2,4,6]. We advised in our patients to avoid sun exposure, use sunscreen regularly, and a moisturizer in case of xerosis. Low-dose oral retinoids were prescribed in 1 patient only.

## Conclusions

Dermoscopy can play a vital role in improving the diagnostic accuracy of EFFC, evade the need for an invasive procedures like skin biopsy, and help us to differentiate it from close mimics. Polarized dermoscopy revealing white rosettes in EFFC further supports the existing literature that rosettes are not specific to any particular condition. They are the result of the optical effect of crossed polarization in the follicular and perifollicular structures.

## Figures and Tables

**Table 1:** Clinico-Demographic Profile and Dermoscopic Findings in Patients of EFFC (n=8)

S.No.	Age	Sex	KP	Sites Involved by Disease							Dermoscopic Findings*							Treatment
				P/A	Cheeks	T/A	SM	Neck	Forehead	Pinna	RBB	Scaling	FP	GD	Ros	Twisted hair	EcV	Oral retinoids**
1	11	M	+	+	+	−	−	−	−	−	+	+	+	+	−	+	+	−
2	12	M	+	+	+	+	−	−	−	−	+	+	+	+	−	+	+	−
3	13	M	+	+	+	+	−	−	−	−	+	+	+	+	−	+	+	−
4	19	F	+	+	+	+	+	+	−	−	+	+	+	+	+	+	+	−
5	15	M	+	+	+	+	+	+	+	+	+	+	+	+	+	+	−	−
6	13	F	+	+	+	−	+	+	−	−	+	+	+	+	−	+	+	−
7	22	M	+	+	+	+	+	+	+	+	+	+	+	+	+	+	−	+
8	24	F	+	+	+	+	+	+	−	−	+	+	+	+	+	+	−	−

* More than one dermoscopic findings were present in each patient

** Oral retinoids were prescribed in 1 patient; other patients were advised to use sunscreen and a moisturizer regularly.

EcV = ectatic vessels; EFFC = erythromelanosis follicularis faciei et colli; FH = family history; FP = follicular papules; GD = gray dots; KP = keratosis pilaris; P/A = preauricular; RBB = reddish brown background; Ros = rosettes; SM = submandibular; T/A = temporal areas.
